# Research on Alleviating Children’s Nighttime Fear Using a Digital Game

**DOI:** 10.3390/children9030405

**Published:** 2022-03-12

**Authors:** Wen Huei Chou, Han-Xing Chen, Ching-Chih Hsu

**Affiliations:** 1 Graduate School of Design, National Yunlin University of Science and Technology, Yunlin 64002, Taiwan; cris@gemail.yuntech.edu.tw (W.H.C.); tinkansei@hotmail.com (H.-X.C.); 2 School of Fine Arts, Nanjing Normal University, Nanjing 210023, China

**Keywords:** nighttime fear, child’s fear, digital game design, adventure game

## Abstract

Nighttime fear is common among children and may negatively affect their growth. Given the positive role of digital games in regulating children’s emotions, in this study, we proposed principles for the design of a digital game to alleviate children’s nighttime fears and developed a game prototype based on a survey of children and their parents. In order to verify whether digital games can reduce children’s fears, the Koala Fear Questionnaire (KFQ) was used to assess the effectiveness of the game prototype in an experiment. We adopted a quasi-experimental design with non-randomized samples, including 47 subjects in the experimental group (EG) and 49 subjects in the control group (CG). The results of the analysis show that the children in the EG displayed an obvious decrease in their fear of the objects that appeared in the game. Moreover, for some children with a moderate level of fear, playing digital games could significantly reduce their fear. Therefore, this preliminary study suggests that digital games have a positive effect on alleviating children’s nighttime fears.

## 1. Introduction

Fear is one of the most common human emotions and an important mechanism of self-defense [1,2]. There are many types of fear, and fear of the night is quite common among children. The incidence of nighttime fear in children aged 7–9 and 10–12 years has reached 84.7% and 79.6%, respectively [3]. After the age of 13, the incidence of children’s nighttime fear declines, but it remains at a high rate [4]. Nighttime fear may lead to children’s inability to concentrate during the day, anxiety [5,6], and a loss of confidence, and could even affect their daily lives [7]. The National Scientific Council on the Developing Child [8] pointed out that, if children are in fear for an extended period, the development of their brain will be adversely affected. Moreover, if children fail to overcome fear in time, they may grow up with fear [9].

With the development of technology, digital games have been used to regulate the emotions of children and adolescents [10], as well as to relieve and transfer their fears [11,12]. Playing digital games properly can also enhance the confidence of young players, which has a positive impact on their psychological health [13]. If digital games can be effectively used to make children feel confident while playing and learning to cope with their own fears, the negative effects of nighttime fears may be reduced accordingly. Therefore, the purpose of this study was (1) to explore the causes and coping methods for local children’s nighttime fears and put forward digital game design principles and solutions to alleviate children’s nighttime fear, and (2) to verify whether digital games can alleviate their nighttime fear.

## 2. Literature Review

### 2.1. The Causes of Children’s Nighttime Fears

Gordon et al. [4] and Muris et al. [3] pointed out that the formation of nighttime fear is related to the characteristics of cognitive development during children’s growth. Some studies have also shown that nighttime fear is related to the negative external information that children receive [14,15] and things that easily trigger fear [1]. According to Rafihi-Ferreira et al. [16], the objects that children fear at night include separation from others (e.g., fear of the death of their parents), personal safety (e.g., the intrusion of kidnappers and thieves), imaginary creatures (e.g., ghosts and monsters), terrible dreams, darkness, and loneliness. Mooney [17] and Muris et al. [3] found that ghosts, monsters, and nightmares are the main things that children aged 4–6 fear at night. When they reach the age of 10–12, the degree of fear of the above-mentioned objects decreases significantly, whereas the fear of bodily injury, physical danger, and intruders increases rapidly. In terms of gender differences, girls reported a higher frequency of nighttime fear [18] and anxiety levels associated with this fear than boys [3]. Gordon’s research found that girls were more afraid of “environmental threats” than boys [18] and are more likely to seek support from their parents [3]. It can be seen that the causes of children’s nighttime fear are affected by their cognitive development level and external negative stimuli, and there are differences in demographic variables such as age and gender, which may affect the results of interventions.

### 2.2. The Methods of Alleviating Children’s Nighttime Fears

In the field of psychology, cognitive-behavioral therapy (CBT) is widely used to alleviate children’s anxiety and fear. This method exposes children to systematic, progressive, and strictly controlled fearful situations and stimuli, so that children become habituated to what they were afraid of, thereby eliminating the fear [19]. Some studies have developed more effective methods to deal with children’s nighttime fears. (1) Bibliotherapy: The study of Lewis et al. [6] used designated reading material with the fear of darkness as the background topic. Parents were required to read this book to their children and play games with them, such as looking for toys in the dark. The results showed that the majority of children participating in the experiment alleviated their fear at night and fell asleep alone more often. Rafihi-Ferreira et al. [16] found that the use of designated reading materials and stuffed dolls representing the protagonists in the books could make it easier for children to immerse themselves in the story and identify with the characters, get through the night without their parents’ company but with the protagonists of the stories, and become trained to sleep alone. The results of the research showed that children’s fear and insomnia improved through the use of these methods, and the incidence of requests for parental companionship decreased. (2) Picture books: Chen [20] found that children engage with the plots and pictures of stories with the theme of the fear of darkness and that they effectively alleviate their fear of darkness. (3) Virtual reality games: A study by Servera, Sáez, and Mir [21] required children with a fear of darkness to explore rooms with varying brightnesses in virtual situations created through VR games and to lie in a dark virtual space for two minutes. As a result, most children achieved satisfactory improvements in terms of their fear. This shows that it is feasible to use games to help children alleviate their fear of darkness. In summary, the existing methods to deal with children’s nighttime fears have the following in common: (a) the use of stories, pictures, or games to create immersive experiences for children to reduce fear; (b) the elimination of the fear of loneliness by increasing the presence of family members or external objects; (c) the setting of specific environments or stimuli that train children to adapt to darkness and nighttime.

### 2.3. The Impact of Digital Games on Children

Children can grow, gain experience and life skills, and learn how to control their emotions and behaviors by playing games [22]. Lee, Xiang, and Gao’s [23] research showed that moderately playing active video games can reduce children’s anger and fear. Furthermore, through the challenges that games pose, children’s cognitive and social skills can be improved [24,25]. In recent years, digital games have been applied in children’s education to improve their learning efficiency and attention [26].

Games are also a type of media used to alleviate children’s fears. Huang et al. [27] incorporated frightening medical processes into live role-playing games, allowing children to complete diagnoses and treatment as the story progressed. An experiment conducted by Windich-Biermeier et al. [28] demonstrated that, compared with books, music, and other media, more children tend to use handheld games to alleviate the fear and pain that intravenous injections cause. DeRosier and Thomas [29] suggested that adventure games are characterized by allowing players to achieve long-term goals through exploration, puzzle-solving, and problem-solving. It can be seen that games affect children via two mechanisms: (a) the sense of immersion in games can divert children’s attention and rid them of painful or fearful situations in reality; (b) games provide difficult situations, which trains children in cognitive learning and problem-solving abilities. Therefore, by allowing children to role-play and go on adventures, digital games can create experiences that allow children to face and overcome their fears.

## 3. Methods

### 3.1. Research Planning

**Figure 1:**
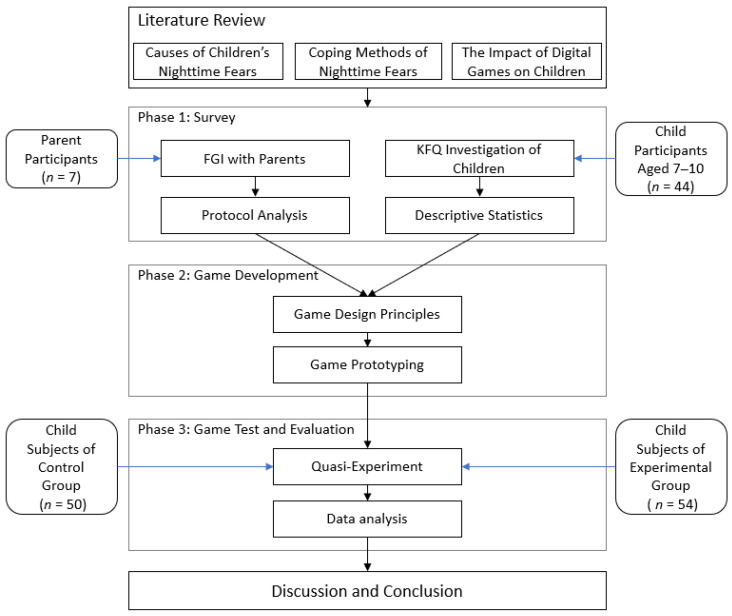
Research phase flow chart.

This study was divided into 3 phases (Figure 1). The purpose of the first phase was to understand the objects and coping methods associated with local children’s nighttime fears. In this study, focus group interviews (FGIs) were used to collect information from parents, and the Koala Fear Questionnaire (KFQ) was used to collect information on children’s perceptions of nighttime fear objects. The second phase transformed the understanding of nighttime fear objects and coping methods obtained in the first phase into game design principles and plans, and the game prototype was developed. The third phase was testing the effect of digital games on alleviating children’s nighttime fears. The test adopted a quasi-experimental design with non-randomized samples, allowing the children in the experimental group (EG) to try the game prototype developed in the second phase, as well as allowing for the collection of the pre-test and post-test data from the EG and the control group (CG). Through an analysis and comparison of the experimental data, we were able to verify the effect of digital games. In order to achieve the research purposes, the following hypotheses were proposed:

**Hypothesis** **1** **(H1).**  *Compared to the children in the CG, the children in the EG will have a significantly lower level of fear after playing the digital game developed in this study.*

**Hypothesis** **2** **(H2).**  *There will be differences in the changes of levels of fear between children of different genders after playing the digital game.*

**Hypothesis** **3** **(H3).**  *There will be differences in the changes of levels of fear among children of different ages after playing the digital game.*

### 3.2. The Focus Group Interview Method

1. Design of the Focus Group Interview Questionnaire

Gordon et al. [4] advocated understanding children’s nighttime fear in three respects, namely, the frequency of nighttime fear, the degree of anxiety, and the object of fear. Since the purpose of this study was to provide solutions for children’s nighttime fear, the FGI questions for parents had to include coping methods. An outline of the FGI questionnaire for parents is shown in Table 1.

2. Participants and the Implementation Process

Muris et al. [3] found that 7–12 years was the age group with the highest incidence of nighttime fear, whereas Stewart and Gordon [30] found that children with severe nighttime fear were aged between 6 and 10 years. The subjects of this study, therefore, comprised children aged 7–10 years. The researcher recruited 7 parents who were willing to accept a visit through social applications to form a focus group. These participants were all parents of children aged 7–10 (Table 2). Affected by the pandemic, the interview was conducted using Microsoft Teams, and it lasted about 50 min. The video documents were converted into verbatim manuscripts for data analysis.

### 3.3. The Koala Fear Questionnaire

(1) Evaluation Criteria and Koala Fear Questionnaire Design

The purpose of this survey was to understand the objects of fear among children in Taiwan. The existing literature includes 17 types of fear objects that could be classified into 6 categories, namely, intruders, fictional creatures, dreams, environmental threats, animals, and fearful thoughts [3,4,16,17]. Taking into account local natural and cultural characteristics, cockroaches, snakes, zombies, etc., were included in this study, and witches and noise were omitted, resulting in 25 items for evaluating children’s nighttime fear (see Table 3).

**Figure 2:**
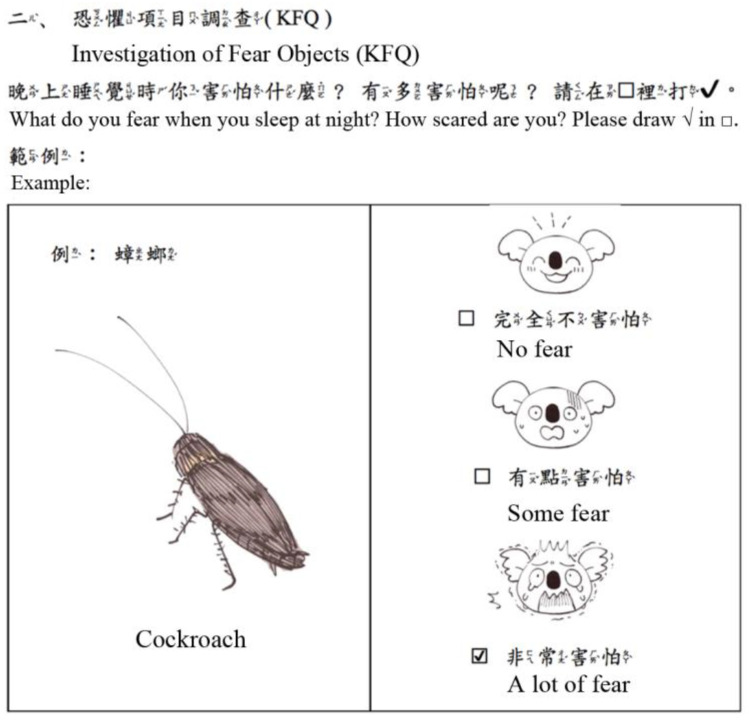
Koala Fear Questionnaire (KFQ) example.

Taking into account the characteristics of children’s cognitive psychological development [30], the KFQ was used as the evaluation tool in this study. The KFQ is a 3-point scale characterized by presenting objects of fear graphically and expressing the degree of fear with a koala showing 3 facial expressions. According to Table 3, the KFQ designed in this research had a total of 25 questions divided into 6 categories. The expressions of some items were adjusted. For example, “worry about the death of parents” was optimized to “separation from parents”. An example of the questionnaire is shown in Figure 2. In addition, there were 3 open-ended questions at the end of the questionnaire: (a) In addition to the above things, what else are you afraid of at night? (b) Why are you afraid? (c) What do you do when you are afraid?

(2) Participants and the Implementation Process

The researchers explained the purpose and significance of the study to primary school students in Southern Taiwan. After the teachers who were gatekeepers signed the informed consent document, the researchers were allowed to issue the KFQs to students aged 7–10 attending the school. A total of 44 questionnaires were returned, and 35 of them were valid. Those who answered valid questionnaires included 13 boys and 22 girls. The age distribution was as follows: 1 people aged 7, 4 people aged 8, 22 people aged 9, and 8 people aged 10.

### 3.4. Experimental Methods

(1) Stimuli and Evaluation Tools

The prototype of the digital game was created based on the literature review, and the results of the survey were used as stimuli in the experiment for the subjects of the EG to experience. To achieve the purpose of this study, the experiment had to be supplemented with tools to evaluate the subjects’ fear before and after playing the game. Therefore, this study used the 25 questions of the KFQ again as the evaluation criteria. Since the KFQ was no longer being used to collect information on fear objects, the last 3 questions of the questionnaire were omitted, and the second KFQ (KFQ-II), which was suitable for the evaluation of experimental effectiveness, was formed.

(2) Participants and the Implementation Process

For studies that intervene in the experience of a particular group, interference factors should be controlled to ensure the homogeneity of the experimental environment, conditions, and subjects. Therefore, the purposive sampling method was adopted, and 2 groups of schoolchildren that were similar in age and gender ratios in 2 primary schools located in the same town were selected as subjects. Since the subjects were not randomly sampled, the experiment in this study was defined as a quasi-experiment. The quasi-experimental process was as follows: First, children in the EG and CG completed the pre-test KFQ-II. Second, the children in the EG entered the computer classroom to play the game for about 30 min, and the children in the CG went to class normally after completing the pre-test KFQ-II for about 30 min. Third, after the children in the EG completed the game trial, the 2 groups of children completed the post-test KFQ-II.

On the premise of obtaining informed consent signed by the parents of the school children, a total of 104 children aged 7–10 participated in the quasi-experiment, including 54 children in the EG and 50 in the CG. Finally, 47 valid questionnaires were collected from the EG and 49 from the CG. The information about the valid samples is shown in Table 4.

## 4. Results of the Surveys

### 4.1. Results of the Focus Group Interviews

By classifying and conceptualizing keywords and phrases in the parents’ verbatim FGI manuscripts, we observed the following three themes:

(a) Children’s sleep habits and needs

When nighttime fear occurs, children show a need for security. First, they asked family members or pets to accompany them. Their behavior includes being unable to fall asleep alone and needing others to accompany them to complete what they could have done independently. The survey also found that children with siblings are less likely to have nighttime fear. Second, children require the creation of a relaxed environment. They expressed their fears and appeals regarding the external environment, such as keeping lights on.

(b) Nighttime fear and its causes

According to the participants, the causes of children’s nighttime fear can be categorized as external or internal. External causes mainly include a fear of fictional creatures and environmental threats, such as ghosts, darkness, earthquakes, and fires. Horror movies and ghost stories are also objects of nighttime fears. These constitute the main causes. The internal causes mainly stem from a lack in the child’s cognition, which produces terrible fantasies and associations.

(c) Coping methods for nighttime fear

Participants mentioned three coping methods for nighttime fear. The first was companionship. Parents gave their children a sense of security by accompanying them when they go to sleep and maintaining physical contact. The second was the assistance of external objects. Night lights, toys, blankets, and other items could increase a child’s sense of security. The third was diverting attention. Talking with children about interesting things or things that they like could reduce anxiety. Most participants believed that the effect of “companionship” was the most obvious among the three.

### 4.2. Results of the Koala Fear Questionnaire

By summing the scores of each item in the 35 valid questionnaires, what children feared the most could be understood by looking at the items with the highest scores. The results showed that, in general, the items that children most feared were kidnappers (84%), death and zombies (both 82%), and thieves (81%). This shows that the fear of intruders accounted for two objects, and the rest were fearful thoughts and fictional creatures. Descriptive statistics also revealed that children of different genders and ages had different fear objects (see Table 5 and Table 6), suggesting that researchers need to verify the effects of different genders and ages on alleviating nighttime fear in follow-up studies.

In response to the extended questions, participants added items that were not listed in the KFQ. Ghosts, skeletons of the dead, and earthquakes appeared most frequently. In fact, the KFQ already had the item of “ghost,” but it may be that the image of the ghost in participants’ imaginations was inconsistent with the picture provided in the KFQ. This result prompted the researchers to attach importance to the influence of fictional creatures on children. The coping methods that participants cited most were seeking their family’s help, hiding, diverting attention, and shouting. These behaviors could be grouped into two categories. The first was seeking help, and shouting could also be understood as a way to seek help. The second category was avoidance. Either hiding or diverting attention was a behavior that could physically or psychologically be expected to remove the object of fear.

In summary, the parents and children’s surveys revealed many similarities. Both groups believed that negative external stimuli, such as fictional creatures, intruders, and environmental threats, are the main factors that trigger children’s nighttime fear. In terms of coping methods, both groups regarded companionship, seeking help, and diverting attention as the most common methods.

## 5. Game Development and Evaluation

### 5.1. Game Design and Development

#### 5.1.1. Design Principles

Game design and development were processes of transforming the information obtained from the literature review and surveys into the setting of game plots, characters, and gameplay. Understanding the causes of nighttime fear inspired the design of the game’s plot. Creating the protagonist had to involve a consideration of the children’s age and identity characteristics; creating obstacles, challenges, and opponent roles took the fear objects as prototypes. Setting the gameplay referred to the methods of coping with nighttime fear. By discerning the relationship between the survey and implementation, we obtained the following design principles:

(a) Use an adventure story as the game’s plot. The literature and surveys showed that nighttime fear is mainly caused by negative information from the external world and the insufficient cognition of children. Therefore, adventure stories should be adopted into the game’s plot so that children can learn while playing to cultivate the ability to overcome fears.
(b) Design roles that meet the characteristics of children. Creating a protagonist who is similar in terms of age and background to the child players makes it easier to identify with the protagonist and immerse themselves into the game’s plot and situation more smoothly. The opponent roles should also take into account children’s physical and mental characteristics to avoid increasing their fear.
(c) Set up challenges and obstacles for the fear objects. The challenges in the game should correspond to the fear objects that the participants described, guide children to face fear objects such as fictional creatures and intruders, and change children’s negative thoughts about these objects into positive thoughts.
(d) Create gameplay that is similar to real-life situations. Concealment, diversion, and help-seeking are all reasonable ways to alleviate fear. In terms of gameplay, if players can drive the characters to try the above methods, they can indirectly learn and master these methods and transfer them to real life.

#### 5.1.2. Development of the Prototype

(a) Game Plot Setting

The game tells the story of a 9-year-old girl who has a nightmare after watching a horror TV show. In her dream, many monsters from the show appear at her home and kidnap her parents. To save her parents, the girl embarks on an adventure. The plot setting utilized the findings of the surveys, that is, that fear stems from negative information from the external world. The introduction of monsters was aimed at responding to objects of fear such as fictional creatures and intruders. The plot also forced children to face the fear of “separation from their parents.”

(b) Game Challenge and Obstacle Design

There were three levels in the game, and the opponents in each level were based on the fear objects that the children mentioned in the questionnaire. According to the survey, the fears that children mentioned most frequently included ghosts, skeletons of the dead, and zombies. To avoid overly realistic content, in this study we implicitly transformed these things into characters that were similar in appearance to a human to create the opponents of the three levels, namely, ghosts, mummies, and zombies.

(c) Role Setting

In addition to the protagonist that the player operated, the game had six opponent characters (partially shown in Table 7). According to the statement of “Protection Level” in Taiwan’s Entertainment Software Rating Management Regulations, game software appropriate for children aged 6–12 can include characters fighting, whereas any content that may cause physical or mental harm to children aged 6 must be avoided. Therefore, although the opponents in our game used the fear objects as their prototypes, during modeling and setting, humorous elements were added, such as ghosts who love to eat honey and zombies who like flowers. Such settings were conducive to directing children’s attention to things that they like and reversing their stereotype of the objects of fear.

(d) Gameplay Setting

**Figure 3:**
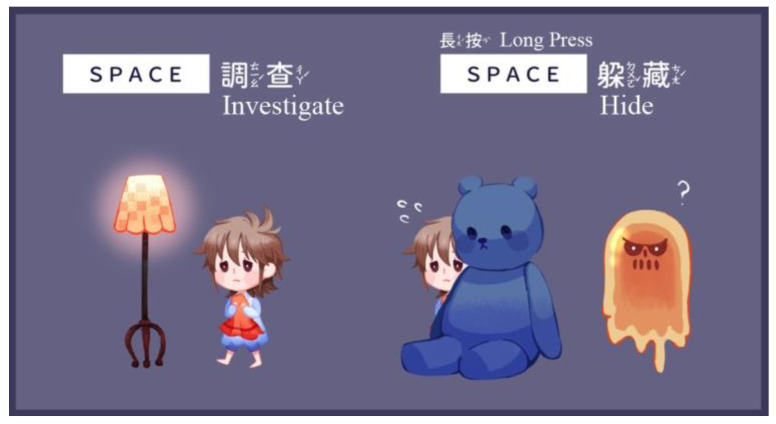
Operation of investigating and hiding.

The results of the survey showed that hiding was the most commonly used coping method for children when encountering fearful events. The game, therefore, incorporated this approach into the gameplay setting (Figure 3). Moreover, puzzle-solving is a characteristic of adventure games. Players need to find, obtain, and reasonably use a variety of props. Numerous elements such as lights and toys were used in the game as props for puzzle-solving and level clearance. These design inspirations were related to the surveys (see Table 8). Notably, in the second level, players needed to help the mother duck find her lost baby, who is an important prop. This reinforced the meaning of overcoming the fear of separation from parents.

### 5.2. Evaluation of the Game’s Effectiveness

#### 5.2.1. Reliability and Validity of the KFQ-II and the Power Analysis

In this research, Cronbach’s α coefficient analysis was used to perform reliability analysis on all observations of each item in KFQ-II. Since there were too few questions for the dimension of “Fear of Dreams” in the original questionnaire, the item “Nightmare” was omitted. Among the remaining five categories of fear objects, the α coefficients of “Intruders”, “Fictional Creatures”, and “Animals” were 0.821, 0.882, and 0.822, respectively, which are all greater than 0.7. The α coefficients for “Environmental Threats” and “Fearful Thoughts” were 0.680 and 0.685, respectively, which are between 0.7 and 0.6 and were thus still within a reliable range. The KFQ is a scale specifically designed to measure fear in children. Compared with other scales that measure fear and anxiety (e.g., the Fear Survey Schedule for Children Revised (FSSC-R) and the State–Trait Anxiety Inventory for Children (STAIC)), the KFQ has high accuracy and effectiveness [31]. Meanwhile, the items in the KFQ-II refer to questions that have appeared in other literature on children’s fears, so this questionnaire has content validity.

In the process of implementing the paired samples difference test for EG, the effect size (Cohen’s d) was measured to be 0.326. According to the calculation conducted using *GPower* software, in the case of α = 0.05 and a one-tailed test, 47 paired samples can theoretically demonstrate a statistical power of 0.711.

#### 5.2.2. Results of the Data Analysis

The mean and standard deviation of the pre-test, post-test, and total scores of the subjects in the EG and CG on the 24 items are shown in Table 9. Due to the quasi-experimental design, all of the subjects in both groups were included based on the class they were in at school, without random sampling, so the sample equivalence had to be tested first. The results comparing the total pre-test scores of the EG and CG through the independent sample *t*-test showed that *p* = 0.342 (>0.05). This indicates that the initial situation and conditions of the two groups were not significantly different, ensuring the internal validity of this quasi-experiment.

Descriptive statistics showed that the mean of the total score of the EG in the post-test was lower than that of the pre-test. Further analysis was performed to calculate the difference between the total scores of the post-test and pre-test in both the EG and the CG, and the independent-samples *t*-test was used to test the two groups in terms of score differences. The results showed that *t* = −2.473 and *p* = 0.016 (<0.05), which indicates significance (see Table 10). In order to understand the changes of the scores of the 24 fear objects in the experiment, the difference between the pre-test and post-test of each item in the EG and the CG was calculated, and the differences were compared one by one with the independent-samples *t*-test. The results showed that 6 of the 24 items exhibited a significant decrease in the post-test scores of the EG compared with the CG. These items include ghosts, monsters, zombies, separation from parents, tigers, and skeletons. The remaining 18 items did not reach a significant level, among which the item “snake” in the EG showed a smaller decline than the CG (*t* = 0.136), whereas the post-test scores of the item “storm” and “the sound of wind blowing” in the EG increased slightly (see Table 10).

**Figure 4:**
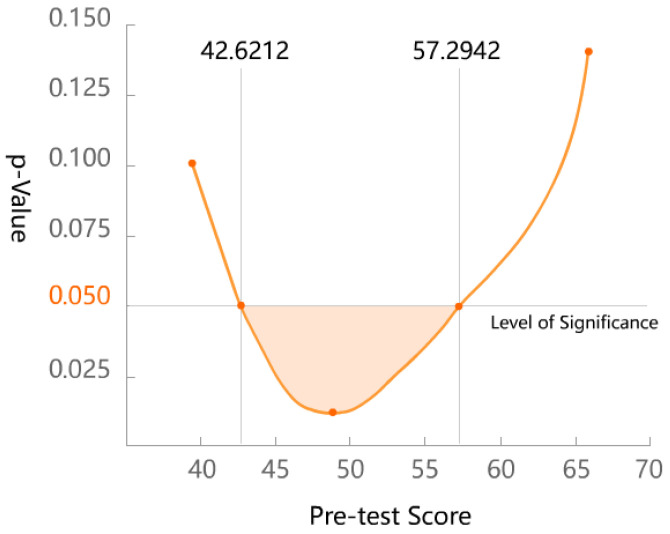
Relationship between the pre-test score and significance.

Observing the total scores of the EG and the CG in the pre-test and post-test, it was found that the scores of both groups decreased in the post-test, but the EG decreased more obviously. The intra-group regression coefficient analysis results of the EG and the CG were F = 7.896, *p* = 0.006 (<0.05), which is significant. This indicated that the regression lines of the two groups were not parallel, which did not meet the basic assumption of ANCOVA. Therefore, the Johnson–Neyman procedure (JNP) was adopted to test the interaction between the grouping variable, the pre-test score, and the post-test score. In the JNP, the grouping variable was defined as the independent variable, the post-test total score was the dependent variable, and the pre-test total score was the covariate. The result showed that, when the pre-test total score was between 42.6212 and 57.2942 points, the post-test scores of the EG and the CG were significantly different (*p* < 0.05), and the EG had a lower score. When the pre-test total score was lower or higher than this interval, there was no significant difference in the post-test scores between the EG and the CG (see Table 11 and Figure 4).

The descriptive statistics revealed that 30 subjects (63.8%) in the EG showed a decrease in the total scores of the post-test. There were five subjects (10.6%) with no change in the total score, including three boys and two girls, four people aged 9, and one person aged 10. There were also 12 subjects (25.5%) who increased in the total score, including three boys and nine girls, two people aged 8, seven people aged 9, and three people aged 10. The increases were mostly between one and three points.

Since there were fewer than 30 male and female subjects in the EG, it is appropriate to implement the method of nonparametric analysis to test whether digital games showed varying effectiveness in terms of gender on alleviating fear. The difference between the total scores of the post-test and the pre-test of male and female subjects in the EG was calculated, and the Mann–Whitney U test was adopted to verify the difference between these two groups. The result showed that *Z* = −1.411, *p* = 0.158. This indicates that the difference between male and female subjects did not reach significance. By sorting the score differences of all subjects from low to high, the Mann–Whitney U test indicated the rank values of male and female subjects. The results showed that the average rank of the male group was 21.00, and that of the female group was 26.64, which indicates that male subjects showed a slightly greater decrease in their scores in the post-test.

To test whether there was a difference in the effectiveness of digital games on alleviating nighttime fears among subjects aged 8, 9, and 10 years, the differences between the total scores of the post-test and the pre-test of the subjects in the three age groups in the EG were calculated. A Kruskal–Wallis one-way ANOVA was used to test the differences of these three groups. The results showed that *p* = 0.248 (>0.05), which is not significant, and there was no need to perform multiple comparisons.

## 6. Discussion and Conclusions

Based on the literature review and surveys of children’s nighttime fears, we put forward four game design principles to guide the setting and prototyping of the plot, roles, levels, and gameplay of digital games to help children to cope with nighttime fears.

On the premise that the initial conditions of the EG and CG were the same, in this study we compared the decline in their scores, and the results show that, in general, the fear of the children in the EG was significantly reduced. Among the 24 items included on the KFQ-II, six items showed a significant decrease, and among these, the items with a significance value lower than or close to 0.01 were ghosts, monsters, zombies, and separation from parents. The four items that appeared in the game were ghosts, mummies, zombies, and separation from parents. It can be seen that three of these showed the most significant decrease, which corroborates the effectiveness of this game.

The results of the JNP test indicated that when the KFQ-II score was between 43 and 57 points, the digital game developed in this study had a significant effect on alleviating children’s fears. With regard to the mean and standard deviation of the subjects’ total scores on the pre-test, the score interval is at the middle level of fear. Among the 96 subjects involved in this study, 52 of them had a total pre-test score between 43 and 57 points, accounting for 54.17% of the total. Therefore, it can be inferred that the game may be effective for some children with a moderate level of fear.

The descriptive statistics showed that the EG contained 12 samples with increased total scores and five samples with no change. Ten of them scored fewer than 43 points or more than 57 points in the pre-test, and two people scored 57 points, which is at the critical point of the game’s effectiveness. The other reason for the increase in scores may have been that the game was more difficult for some children, so their fears were not alleviated while playing. In addition, among the 24 items, the total score of 22 decreased, but the scores for “Storm” and “Sound of wind blowing” increased. We speculated that the game used in the experiment did not involve obviously related stimuli, and the environmental threat was different from the fear objects that could be easily represented. That is, environmental fear mainly existed in the game in the background, so it was difficult to detect and associate it with the fear objects.

Although the findings of this research do not support the claim that subjects of different ages and genders have significant differences in terms of the effectiveness of the game on fear alleviation, the results of the Mann–Whitney U test indicated that boys were more affected than girls. Descriptive statistics also showed that, among the 12 samples in the EG with an increase in total scores, nine were female, accounting for 36% of all female subjects. In terms of age, 30% of the 8- to 9-year-old subjects showed an increase in their post-test scores, which indicated that the effect of fear alleviation for 10-year-old subjects was stronger than that for 8- to 9-year-old subjects. Furthermore, behavior observations during the experiment showed that, compared with male subjects, females were more likely to be confused about the operation of the game. In addition, the skills and flexibility of subjects from lower grades in operating computer games were generally weaker than those of subjects from higher grades. This study, therefore, leads us to speculate that the ease of use of game software and equipment affects the alleviation of users’ fears.

Therefore, we propose the following suggestions for improving the game design based on the experimental results:

(a) Providing easier gameplay for children in lower grades. The game used in this experiment was played on desktop computers and was operated mainly using a keyboard. Therefore, reducing the number of keys required and reducing the combinations of keys would make the operation smoother for young children. Future experiments should also consider the application of more familiar and simpler equipment for children.
(b) Ensuring that the content of the game is more in line with children’s intuitive thinking. In future game design, the first consideration should be whether the game’s logic is consistent with the intuitive associations of young children. If the props and challenges are set logically, it will help children to understand and improve level clearance. Second, concretizing and personifying abstract things may be more in line with children’s intuitive thinking and may draw their attention to and increase their associations among such things, thus improving the effectiveness of alleviating fear.
(c) Providing diversified media to increase immersion in the game. In accordance with the literature, the game used in this study tells its story in the form of a picture book. If the variety of media used can be increased, e.g., by including audio descriptions, background music, sound effects, and animation, it will improve children’s attention and efficiency in receiving information, allowing them to more smoothly immerse themselves in the atmosphere and situation that the game creates.

Due to the influence of the pandemic and the limitation of the study’s scale, a cross-sectional approach and a quasi-experimental design with non-randomized samples were adopted. Therefore, the findings and conclusions of this preliminary study are exploratory and tentative with regard to the issue of using digital games to moderate children’s fears. In addition, the place, environment, and time of the experiment were different from real situations in which nighttime fear occurs. Therefore, using a true experimental design with random samples, increasing the sample size, and providing a more realistic experimental environment could make the research more rigorous and convincing in order to obtain more effective and practical approaches to alleviate children’s nighttime fears.

## Figures and Tables

**Table 1:** Outline of the focus group interview (FGI) for parents.

Dimension	Question
Children’s sleep situation	1. Can your children fall asleep alone? 2. Can your children sleep alone without light and sound? 3. How often does nighttime fear occur, and how long does it last?
The causes of nighttime fear and the degree of anxiety	1. Do you know what children fear when they cannot sleep? 2. Do you know how this fear arises? 3. How serious is the child’s anxiety level when facing fears? What behaviors do they have?
Coping methods	1. How do you help children when they cannot sleep alone? 2. What is the effect of the above coping methods?

**Table 2:** Interviewee information.

Code	Gender	Age	Kinship	Age of Child	Gender of Child	Sibling
A	F ^1^	35	Mother	8	M	Yes
B	F	35	Mother	7	F	Yes
C	F	35	Mother	7	F	Yes
D	F	42	Mother	9	M	Yes
E	F	39	Mother	7	F	Yes
F	F	31	Aunt	10	M	No
G	M ^2^	39	Father	7	M	Yes

^1^ F = Female; ^2^ M = Male.

**Table 3:** Children’s nighttime fear objects.

Category	Fear Object
Intruder	Thieves, kidnappers
Fictional creatures	Ghosts, monsters, zombies, vampires, mummies, skeletons of the dead, dinosaurs
Dreams	Scary dreams
Environmental threats	Darkness, storms, the sound of knocking, the sound of wind blowing, shadows in the room
Animals	Spiders, dogs, lions, tigers, snakes, cockroaches, hornets
Fearful thoughts	Threats to personal life, fear of parents’ death, loneliness

**Table 4:** Information on the subjects.

Group	*N*	Gender		Age (Year)		
		Male	Female	8	9	10
EG ^1^	47	22	25	4	26	17
CG ^2^	49	27	22	8	27	14

^1^ EG = Experimental Group; ^2^ CG = Control Group.

**Table 5:** Ranking of fear objects for different genders.

Gender	Rank 1	Rank 2	Rank 3
Male	Death; separation from parents (79.5%)	Thief (76.9%)	Kidnapper; zombie (74.4%)
Female	Kidnapper (89.4%)	Zombie; mummy (86.4%)	Hornet (85.8%)

**Table 6:** Ranking of fear objects for different ages.

Age (Year)	Rank 1	Rank 2	Rank 3
8	Death (100%)	Falling asleep alone (91.7%)	Kidnapper; thief; skeleton of the dead; hornet (83.3%)
9	Zombie (89.4%)	Mummy (86.4%)	Kidnapper (84.8%)
10	Thief; spider; death (87.5%)	Kidnapper (83.3%)	Snake; hornet (79.2%)

**Table 7:** Partial character settings.

Role	Introduction
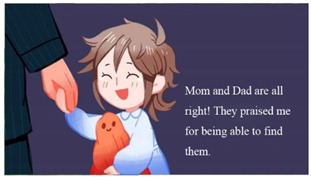 Main Character	A player-operated character with the ability to jump, explore, and hide.
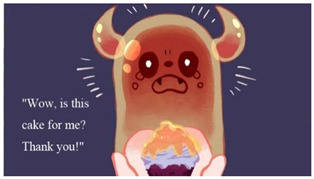 Ghost King	He likes sweets and hates bitterness and can be beaten with coffee.
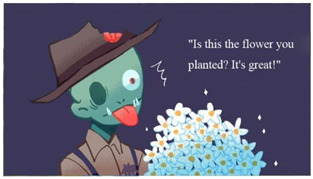 Zombie King	He likes gardening. Helping him to grow flowers ends the game.

**Table 8:** Partial props.

Prop	Gameplay
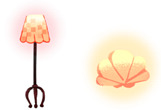 Lights	This allows players to see their surroundings so that they can explore.
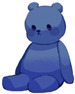 Bear Doll	Players can hide behind this bear to avoid injury.
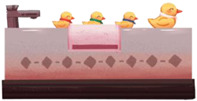 Mother Duck and Baby Ducks	Players need to find the third baby duck and return it to the mother duck to obtain the key prop.

**Table 9:** Mean and standard deviation of experimental data.

Item	Pre-Test for the EG ^1^	Post-Test for the EG	Pre-Test for the CG ^2^	Post-Test for the CG
1. Kidnapper	2.32 (0.747)	2.09 (0.794)	2.37 (0.691)	2.14 (0.700)
2. Thief	2.15 (0.771)	2.02 (0.812)	2.41 (0.697)	2.33 (0.711)
3. Ghost	2.21 (0.898)	1.64 (0.810)	1.98 (0.769)	1.94 (0.793)
4. Monster	1.94 (0.783)	1.60 (0.762)	1.78 (0.789)	1.82 (0.825)
5. Zombie	2.51 (0.768)	1.98 (0.887)	2.14 (0.782)	2.06 (0.793)
6. Vampire	1.94 (0.861)	1.66 (0.806)	2.20 (0.857)	2.06 (0.818)
7. Mummy	1.96 (0.898)	1.81 (0.816)	1.94 (0.767)	1.94 (0.867)
8. Skeleton	2.15 (0.875)	1.94 (0.885)	1.82 (0.849)	1.90 (0.931)
9. Dinosaur	1.49 (0.740)	1.40 (0.704)	1.61 (0.803)	1.67 (0.766)
10. Darkness	1.72 (0.791)	1.62 (0.759)	1.69 (0.788)	1.63 (0.800)
11. Storm	1.40 (0.641)	1.47 (0.680)	1.16 (0.370)	1.20 (0.403)
12. Sound of knocking	1.77 (0.778)	1.70 (0.741)	1.43 (0.571)	1.43 (0.639)
13. Sound of wind blowing	1.32 (0.510)	1.34 (0.517)	1.33 (0.549)	1.29 (0.452)
14. Shadow in the room	1.57 (0.792)	1.55 (0.738)	1.55 (0.672)	1.55 (0.702)
15. Dog	1.72 (0.817)	1.62 (0.759)	1.67 (0.711)	1.78 (0.736)
16. Lion	1.91 (0.767)	1.83 (0.833)	2.22 (0.763)	2.16 (0.765)
17. Tiger	2.00 (0.825)	1.83 (0.833)	2.18 (0.747)	2.27 (0.750)
18. Snake	2.19 (0.762)	2.13 (0.789)	2.31 (0.676)	2.22 (0.763)
19. Spider	2.23 (0.831)	2.19 (0.866)	1.90 (0.789)	1.92 (0.778)
20. Cockroach	2.21 (0.797)	2.13 (0.815)	1.92 (0.829)	1.84 (0.841)
21. Hornet	2.57 (0.574)	2.45 (0.646)	2.24 (0.821)	2.14 (0.756)
22. Death	2.55 (0.612)	2.32 (0.747)	2.39 (0.828)	2.22 (0.863)
23. Separation from parents	2.47 (0.739)	2.11 (0.805)	2.35 (0.797)	2.24 (0.796)
24. Sleep alone	2.00 (0.825)	1.79 (0.849)	1.69 (0.788)	1.63 (0.720)
Total Score	48.32 (11.620)	44.19 (13.010)	46.29 (8.734)	45.39 (9.795)

^1^ EG = Experimental Group; ^2^ CG = Control Group.

**Table 10:** Paired samples *t*-test of items in the experimental group and the control group.

Item	*t*	*p*	Item	*t*	*p*
1. Kidnapper	−0.082	0.935	13. Sound of wind blowing	0.678	0.499
2. Thief	−0.350	0.727	14. Shadow in the room	−0.162	0.872
3. Ghost	−3.654	0.000 *	15. Dog	−1.906	0.060
4. Monster	−3.150	0.002 *	16. Lion	−0.220	0.826
5. Zombie	−3.152	0.002 *	17. Tiger	−2.331	0.022 *
6. Vampire	−1.005	0.318	18. Snake	0.136	0.892
7. Mummy	−1.032	0.305	19. Spider	−0.688	0.493
8. Skeleton	−2.263	0.026 *	20. Cockroach	−0.033	0.974
9. Dinosaur	−1.151	0.253	21. Hornet	−0.223	0.824
10. Darkness	−0.342	0.733	22. Death	−0.524	0.601
11. Storm	0.253	0.801	23. Separation from parents	−2.528	0.014 *
12. Sound of knocking	−0.580	0.563	24. Sleep alone	−1.498	0.138
Total score	−2.473	0.016			

* Significance reached (*p*-value < 0.05).

**Table 11:** Conditional effect of group on the post-test at values of the pre-test.

Pre-Test Score	Effect	*p*-Value
24.0000	−1.7145	0.6043
30.7500	−2.1228	0.3995
35.2500	−2.3949	0.2404
42.6212	−2.8408	0.0500 *
48.7500	−3.2115	0.0174 *
51.0000	−3.3476	0.0194 *
57.2942	−3.7282	0.0500 *
60.0000	−3.8919	0.0731
64.5000	−4.1641	0.1182
69.0000	−4.4362	0.1647

* Significance reached (*p*-value < 0.05).

## Data Availability

Raw data supporting reported results can be obtained from the corresponding author upon reasonable request.
